# Self-Medication Pattern among Social Science University Students in Northwest Ethiopia

**DOI:** 10.1155/2017/8680714

**Published:** 2017-01-16

**Authors:** Dessalegn Asmelashe Gelayee

**Affiliations:** Department of Pharmacology, College of Medicine and Health Sciences, University of Gondar, Gondar, Ethiopia

## Abstract

*Background*. Inappropriate self-medication causes wastage of resources among others.* Method*. This survey study was conducted to determine self-medication pattern of 404 social science university students in Northwest Ethiopia, who were selected through stratified random sampling technique. Data were collected using self-administered questionnaire and analyzed with SPSS version 20 statistical software. Binary Logistic Regression analysis was employed with *P* value < 0.05 considered statistically significant.* Result*. At 95.3% response rate, mean age of 21.26 ± 1.76 years, and male/female ratio of 1.26, the prevalence of self-medication during the six month recall period was 32.7%. Headache (*N* = 87, 69.1%) was the primary complaint that prompted the practice and hence analgesics (*N* = 67, 53.2%) were the mostly used drugs followed by antimicrobials (*N* = 50, 39.7%). The top two reasons driving the practice were nonseverity of the illness (*N* = 41, 32.5%) and suggestions from friends (*N* = 33, 26.2%). Female sex (*P* = 0.042) and higher income (*P* = 0.044) were associated with the practice.* Conclusion*. Self-medication practice, involving the use of both nonprescription and prescription drugs such as antimicrobials, among the social science university students is high. Therefore health education interventions regarding the risks of inappropriate self-medication are essential.

## 1. Introduction

Illness or symptoms of an illness are a common human experience for which the actions taken vary depending on the perceptions and experiences of individuals and other factors. Self-care is the major form of care in illness, which is the oldest and most widely used behavior that affects the health of individuals [1]. Self-medication is the selection and use of medicines by individuals to treat self-recognized illnesses or symptoms. Recognition of the responsibility of individuals for their own health and the awareness that professional care for minor illness is often unnecessary have contributed to the concept of self-medication. A responsible self-medication involves the use of medicines which are approved and available without prescription and which are safe and effective when used as directed [2]. The practice however also involves the use of herbal medicines and prescription only drugs such as antibiotics [3, 4]. Acquiring medicines without a prescription, resubmitting old prescriptions to purchase medicines, sharing medicines with relatives or members of one's social circle, or using leftover medicines stored at home is considered as self-medication practice [5].

This practice has been on the rise worldwide with huge variation in its prevalence among developing and developed nations due to inherent differences in cultural and socioeconomic factors and disparities in health care systems such as reimbursement policies and access to healthcare and drug dispensing policies [6]. Different factors at individual level such as age, sex, income, self-care orientation, education level in general and medical knowledge in particular, access to drugs, and exposure to advertisements also influence the practice [7, 8].

In economically deprived countries most episodes of illness are treated by self-medication [9] imposing much public and professional concern about the irrational use of drugs [10]. A lot has been done on improving access to essential drugs and rational drug use including self-medication practice has been a subject of interest. Medical care in Ethiopia is largely based on out of pocket expenditure of the patients and there are student clinics where the care is given to college students for free. In the nation, few studies have been carried on self-medication practice of the general public as well as among healthcare university students. However data on social science university students is unavailable. It is assumed that the pattern of self-medication may differ in these populations as their curriculum is devoid of medical training. Hence, to make tailored interventions, it is important to characterize the problem in such population. In addition, investigating self-medication among tertiary level students is important as they constitute a segment of the society that is highly educated and more inclined to information about health [4]. This study was therefore carried out to assess the pattern of self-medication among social science students of University of Gondar, Northwest Ethiopia.

## 2. Material and Methods 

A cross-sectional study was conducted from February to June 2014 at University of Gondar, Northwest Ethiopia, which at the time had five campuses. One of them, Maraki Campus, was selected for the study because of convenience for data collection. There were a total of 5685 social science students during the study. The sample size was determined using a formula of *n* = *z*^2^*P*  (1 − *P*)/*w*^2^ and 5% contingency with the following assumptions: a *P* value of 0.05, *z* = 1.96 and CI = 95%, and *w* = 0.05. Thus 404 respondents were chosen with a stratified random sampling technique based on seniority and sex. The inclusion criterion was enrollment in one of the social science programs in the campus during the study period. Ethical approval was obtained from the Department of Pharmacology, College of Medicine and Health Science, University of Gondar, and then data was collected using a pretested self-administered questionnaire with both open and closed ended questions. The instrument was adapted from previous similar studies [5–7] and had items regarding sociodemography as well as pattern of self-medication. Finally, the collected data were checked for completeness and entered to SPSS version 20 statistical software for further analysis using Binary Logistic Regression with *P* value < 0.05 taken as statistically significant.

## 3. Results

Among the four hundred and four participants, 385 completed the questionnaire making a 95.3% response rate. Most of the respondents were males (55.8%), third-year students (33.8%), and with an average monthly middle income of 200–500 Eth birr (64.7%). The mean age ± SD of the respondents was 21.26 ± 1.76 years (Table 1).

As shown in Table 2, one hundred and twenty-six (32.7%) respondents practiced self-medication within the 6-month recall period preceding this study. The majority of them (40.5%) did the practice more than twice and 22.2% respondents described that their self-medication practice extended for a period of one week up to a month. Analgesics were the most commonly used drugs (*N* = 67, 53.2%) followed by antimicrobials (*N* = 50, 39.7%), antacids (10.3%), and vitamins (8.7%).

**Figure 1:**
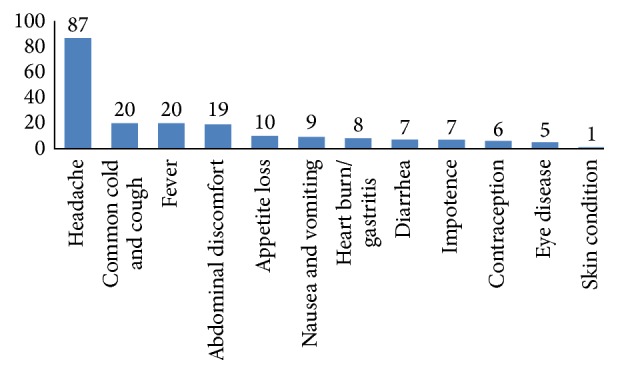
Medical conditions for which self-medication was practiced (*N* = 126). Note: it is multiple response questions.

The main medical conditions that prompted self-medication were headache (*N* = 87, 69.1%), common cold (*N* = 20, 15.9%), fever (*N* = 20, 15.9%), and abdominal discomfort (*N* = 19, 15.1%) (Figure 1). Mildness of the disease (32.5%), suggestions of friends (26.2%), and inexpensiveness of the practice (25.4%) were the top three driving reasons (Table 3).

In the present study, the practice of self-medication was significantly associated with female sex (AOR = 1.658, CI [1.020–2.696], *P* = 0.042) and higher income (AOR = 2.153 CI [1.020–4.545], *P* = 0.044) (Table 4).

## 4. Discussion

The study population in this cross-sectional study was social science students of University of Gondar, Northwest Ethiopia. They were young people of similar age group and academic backgrounds. The 32.7% prevalence of self-medication practice found in the present study is relatively higher than the 27.2% prevalence among the general public in and around the study area reported in a previous study [11]. The difference might be due to increased academic status of the respondents in this study as the positive impact of literacy on self-medication practice is documented in previous studies and the ratio of literate people who are practicing self-medication is high as compared to those of illiterate people [12]. However, the prevalence in the present study is relatively lower than 38.5% [13], 43.24% [14], and 45.89% [15] prevalence reports in studies carried out in Ethiopia. The medical training background of the respondents in the latter three studies may explain the difference because such training was shown to influence self-medication by students in other studies [16]. On the other hand the prevalence of self-medication practice in this study varies inconsistently with studies done abroad among university students: 19% in Portugal [17], 59% in United Arab Emirates [18], 75.7% in India [19], 86.4% in Brazil [20], and 92.3% in Slovenia [7]. The difference might arise due to variation in the socioeconomic factors and sociodemographic characteristics as well as the methodologies used to assess self-medication practice.

While appropriate and responsible self-medication practice is good in that it can save time and readily relieve acute health problems and may save life in such serious conditions and may be economical too for the individual as well as the health care system [2, 21], it can however also bring several harms when practiced inappropriately. With wrong self-medication practice resources are wasted, drug resistance of pathogens is increased, adverse drug reactions happen, drug dependency develops, and the health suffering increases [21, 22]. In relation to this, irrational use of drugs is a huge global concern with half of the medicines worldwide being prescribed, dispensed, or sold irrationally and 50% of patients failing to adhere to the prescribers' advice and not taking them correctly [23]. In the face of such challenges, more frequent and extended self-medication practice among a population of individuals without medical background may be inappropriate. In the present study, the respondents were engaged in more frequent (more than twice for 40.5% respondents) and more extended self-medication practice (for longer than a week by 31% of respondents who did self-medication). Such pattern of self-medication in the 6-month recall period may be associated with too many problems described above and it needs appropriate interventions.

Several previous studies have shown that headache, fever, gastrointestinal disease, and respiratory tract infection are some of the common medical conditions that prompted self-medication [14, 19, 24, 25]. Similarly in this study headache was the top first condition that prompted self-medication and hence analgesics were the mostly used drugs. Another study showed that headache is highly prevalent (as high as 87.7%) among university students [26].

In the present study, headache was followed by common cold and cough and fever as well as abdominal discomforts which is very similar to previous studies [13, 15, 17, 20, 24, 27].

Antimicrobials were the second mostly used drugs (39.68%) during self-medication and this should be considered an alarming problem as misuse of these drugs is a threat to development of drug resistance. Inappropriate use of antimicrobials is known to be common in the developing countries where there is increased access to such drugs without a prescription [28]. Paralleling this, a high prevalence of self-medication with antibiotics among college students is reported in Ghana (70%) [4] and Iran (53%) [29]. In the current study using antimicrobials for self-medication is however higher (more than twice) than that reported by Abay and Amelo [13] among healthcare university students in the same area few years back. The difference may arise from decreased awareness of drug resistance in the social science students of this study and accordingly they might be more likely to be engaged in indiscriminate use of antimicrobials for self-medication practice.

The nonseverity of the health problem, previous experience, and friends' suggestion as well as inexpensiveness were identified to be the reasons for practicing self-medication in this study. It is consistent with previous studies conducted among university students as well as the general public [4, 5, 13, 14, 18, 25, 27, 30]. However such reasons may sometimes be unacceptable as treatment on the bases of previous experience may result in misdiagnosis and wrong choice of drugs since diseases may share similar symptoms. Therefore incorrect treatment of even mild problems may prolong the suffering or may worsen the problem and this is likely because the respondents are social science students and lack medical background which enhances disease diagnosis. In line with this, previous study in Ethiopia reported that 55.4% of those who practiced self-medication admitted deterioration of their condition following self-medication [31]. The present study however identified reasons for self-medication which are not commonly reported so far such as lack of trust on the healthcare professionals as well as easy access to drugs. The unregulated drug dispensing practice which is a feature of developing countries may be associated with the latter reason.

In this study, sex (being female) encouraged self-medication practice (*P* = 0.042) and is in agreement with previous studies [6, 14]. It may be due to higher medication sharing practice among females compared to males which encourages self-medication practice. This however needs to be further studied. High monthly income (>500 Eth Birr) was also shown to encourage self-medication practice (*P* = 0.044) which may be related to high purchasing power as reported in previous studies [32]. However seniority in educational level did not significantly influence self-medication practice (*P* > 0.05) unlike that of Osemene and Lamikanra [6], Klemenc-Ketis et al. [7], Abay and Amelo [13], and Gutema et al. [14]. Lack of medical training for the respondents in this study may explain the difference as seniority did not add any medical knowledge in contrary to respondents in the other studies.

## 5. Conclusion

There is high prevalence of self-medication practice among the social science university students including the use of antimicrobials. This is significantly influenced by sex and income of the respondents. Therefore health education intervention regarding the risks of inappropriate self-medication is essential.

## Figures and Tables

**Table 1:** Sociodemography of respondents.

Sociodemographic characteristics	Frequency	Percent (%)
Sex		
Male	215	55.8
Female	170	44.2
Year of study		
First year	116	30.1
Second year	113	29.4
Third year	130	33.8
Fourth year	12	3.1
Fifth year	14	3.6
Monthly income		
Less than 200 Eth birr	80	20.8
200–500 Eth birr	249	64.7
Greater than 500 Eth birr	56	14.5

1USD = 19.95 Eth birr.

**Table 2:** Prevalence, frequency, and duration of self-medication practice.

Variable	Response	
	Number of students	(%)
Did SM in the past 6 months (*N* = 385)		
Yes	126	32.7
No	259	67.3
Frequency of SM in the past 6 months (*N* = 126)		
Once	42	33.3
Twice	33	26.2
More than twice	51	40.5
Duration of SM (*N* = 126)		
For <1 week	87	69.1
For 1 week-1 month	28	22.2
For >1 month	11	8.7

Note: SM = self-medication.

**Table 3:** Reasons for practicing self-medication (*N* = 126).

Reasons	Frequency	(%)
Mildness of the problem	41	32.5
Friends' suggestion	33	26.2
Self-medication is cheaper	32	25.4
Previous experience	25	19.8
Do not trust health professionals	20	15.9
Obtaining drugs easily	20	15.9
Being embarrassed to tell about disease	10	7.9
Long waiting time	9	7.1
Long distance from health facility	4	3.2
Can afford cost of drugs	3	2.4

Note: it is multiple response question.

**Table 4:** Determinants of self-medication practice.

Variable	Number of students who did self- medication		Crude Odds ratio	Adjusted odds ratio	*P* value
	Yes	No			
Sex					
Male	64	151	1	1	
Female	62	108	1.354	1.658 [1.020–2.696]	0.042^**∗∗∗**^

Monthly income (Eth. birr)					
<200	27	53	1	1	
200–500	67	182	0.723	0.661 [0.371–1.176]	0.159
>500	32	24	2.617	2.153 [1.020–4.545]	0.044^**∗∗∗**^

Year of study					
First year	38	78	1	1	
Second year	29	84	0.709	0.782 [0.427–1.432]	0.426
Third year	45	85	1.087	1.154 [0.633–2.105]	0.640
Fourth year	7	5	2.874	2.977 [0.818–10.841]	0.098
Fifth year	7	7	2.053	1.819 [0.546–6.058]	0.330
